# Huntington disease iPSCs show early molecular changes in intracellular signaling, the expression of oxidative stress proteins and the p53 pathway

**DOI:** 10.1242/dmm.019406

**Published:** 2015-09-01

**Authors:** Wojciech J. Szlachcic, Pawel M. Switonski, Wlodzimierz J. Krzyzosiak, Marek Figlerowicz, Maciej Figiel

**Affiliations:** Institute of Bioorganic Chemistry, Polish Academy of Sciences, Noskowskiego 12/14, Poznań 61-704, Poland

**Keywords:** Huntington disease, MAPK pathway, SOD1, Wnt pathway, iPS, iPSC, p53 pathway

## Abstract

Huntington disease (HD) is a brain disorder characterized by the late onset of motor and cognitive symptoms, even though the neurons in the brain begin to suffer dysfunction and degeneration long before symptoms appear. There is currently no cure. Several molecular and developmental effects of HD have been identified using neural stem cells (NSCs) and differentiated cells, such as neurons and astrocytes. Still, little is known regarding the molecular pathogenesis of HD in pluripotent cells, such as embryonic stem cells (ESCs) and induced pluripotent stem cells (iPSCs). Therefore, we examined putative signaling pathways and processes involved in HD pathogenesis in pluripotent cells. We tested naïve mouse HD YAC128 iPSCs and two types of human HD iPSC that were generated from HD and juvenile-HD patients. Surprisingly, we found that a number of changes affecting cellular processes in HD were also present in undifferentiated pluripotent HD iPSCs, including the dysregulation of the MAPK and Wnt signaling pathways and the dysregulation of the expression of genes related to oxidative stress, such as *Sod1*. Interestingly, a common protein interactor of the huntingtin protein and the proteins in the above pathways is p53, and the expression of p53 was dysregulated in HD YAC128 iPSCs and human HD iPSCs. In summary, our findings demonstrate that multiple molecular pathways that are characteristically dysregulated in HD are already altered in undifferentiated pluripotent cells and that the pathogenesis of HD might begin during the early stages of life.

## Abstract (precis)

**Summary:** This research demonstrates that dysregulation of signaling pathways is a very early event in the pathogenesis of Huntington disease and that these pathways are already dysregulated in cells at the stage of pluripotency.

## INTRODUCTION

Huntington disease (HD) is a hereditary dominant neurodegenerative disorder evoked by the excessive expansion of the CAG repeats in the *HTT* gene (The Huntington's Disease Collaborative Research Group, 1993). The mutant *HTT* allele produces mutant huntingtin protein containing a long polyglutamine region, which evokes pathological changes in cellular physiology resulting in neuronal death and the degeneration of neuronal networks within the brain. Pathologic changes in neuronal brain cells, for instance in the cerebral cortex and striatum, elicit the development of chorea and cognitive impairments and lead to earlier patient death (Papoutsi et al., 2014; Roos, 2010).

Although the symptoms of HD appear relatively late, during the third and fourth decade of life, the impact of the presence of mutant and normal huntingtin can be identified substantially earlier. First, HD can be detected in affected individuals decades before the classical neurological diagnosis is reached (Paulsen et al., 2008). In both affected humans and HD mouse models, neuronal degeneration in the cortex and striatum and dysregulation of neurodevelopmental pathways in these brain structures occurs long before the onset of classical HD symptoms and phenotypic changes (Cummings et al., 2006; Hodges et al., 2006; Kuhn et al., 2007; Milnerwood et al., 2006; Schippling et al., 2009). In addition, the presence of huntingtin is required during the early stages of embryogenesis and during differentiation (Conforti et al., 2013; Duyao et al., 1995; Woda et al., 2005; Zeitlin et al., 1995; Nasir et al., 1995). Mutant huntingtin can induce the precocious specification of a neuroectodermal fate (Nguyen et al., 2013). In HD mouse models, stem-cell-mediated neurogenesis in the striatum and the deployments of the core pluripotency factors SOX2 and NANOG are impaired (Molero et al., 2009). Similarly, the development of the cerebral cortex is impaired in HD mice (Molina-Calavita et al., 2014). In summary, several studies have suggested that the pathogenic process of HD might start early in life, and it has been postulated for some time that the pathological processes might develop successively, starting at birth (Penney et al., 1997). The questions that remain are how early the molecular and functional changes related to HD occur, and which of them occurs first.

Recently, an HD phenotype was demonstrated in HD neural stem cells (NSCs) and HD neuronal cells that were differentiated from patient induced pluripotent stem cells (iPSCs) (An et al., 2012; Mattis et al., 2015; The HD iPSC Consortium, 2012). In addition, treating iPSCs with a proteasome inhibitor induced the production of HD inclusions (Jeon et al., 2012). Moreover, iPSCs generated from R6/2 mice showed defects in the expression of proteins involved in lysosomal formation, cholesterol synthesis and pluripotency (Castiglioni et al., 2012). Therefore, we investigated the HD pathogenic processes and markers using both HD YAC128 iPSCs that we generated and human HD iPSC lines. Both cell types showed a number of dysregulated cellular processes, indicating that these events might be among the earliest markers of HD. The dysregulated processes included ERK signaling, β-catenin phosphorylation, SOD1 accumulation and the expression of p53, which is a common interactor of ERKs and GSK3β. In summary, we demonstrate that a host of key HD pathogenic processes are active under the embryonic stem cell (ESC)-like cellular conditions that are characteristic of iPSCs. Our data indicate that the pathogenesis and alteration of signal transduction in HD begins in the early stages of life.

**TRANSLATIONAL IMPACT**

**Clinical issue**

Huntington disease (HD) is one of several genetic neurodegenerative diseases that are caused by the expansion of trinucleotide CAG repeats in certain causative genes (the huntingtin gene in the case of HD). The classical motor and cognitive symptoms of HD are associated with the expression of mutant huntingtin mRNA and protein, which are particularly toxic for neurons, and occur late, during the third and fourth decades of life. Interestingly, some alterations, such as neuronal degeneration and dysregulation of neurodevelopmental pathways, are detectable in both human patients and HD cell and mouse models long before classical HD symptoms manifest. This suggests that the disease process begins substantially earlier than indicated by the onset of classical HD symptoms. The questions that remain are how early in the disease process the molecular and functional changes related to HD occur, and which of them occurs first. Recent data indicate that transcription factors and other proteins involved in intracellular cascades might be responsible for the early disease alterations seen in HD.

**Results**

In this study, to identify early markers of HD, the authors used human HD-induced pluripotent stem cells (iPSCs) from HD- and juvenile-HD-affected individuals, and generated a model of undifferentiated naïve cells (HD YAC128 iPSCs). These cells expressed mutant huntingtin and resembled embryonic stem cells (ESCs). None of the tested cells contained reprogramming factors that could affect the occurrence of non-specific signs of HD. The authors discovered that some key molecular pathways, including the ERK1/2 and Wnt pathways, certain oxidative-stress-related genes and p53 are dysregulated in HD iPSCs. These results suggest that dysregulation of such signaling pathways is a very early event in the pathogenesis of HD and that these alterations occur in cells at the stage of pluripotency.

**Implications and future directions**

Detecting early molecular signs of HD already in pluripotent cells can change and advance our understanding of the onset of HD, which is typically regarded as an age-related disease. The current data indicate that the HD presymptomatic stage is characterized by early molecular processes that might lead to the symptomatic stage of the disease. This study also identified candidate molecules that can be further investigated as potential targets for early protective therapeutic approaches and for validating anti-HD drugs.

## RESULTS

### Generation of iPSCs using skin fibroblasts isolated from the YAC128 HD model

**Fig. 1.**
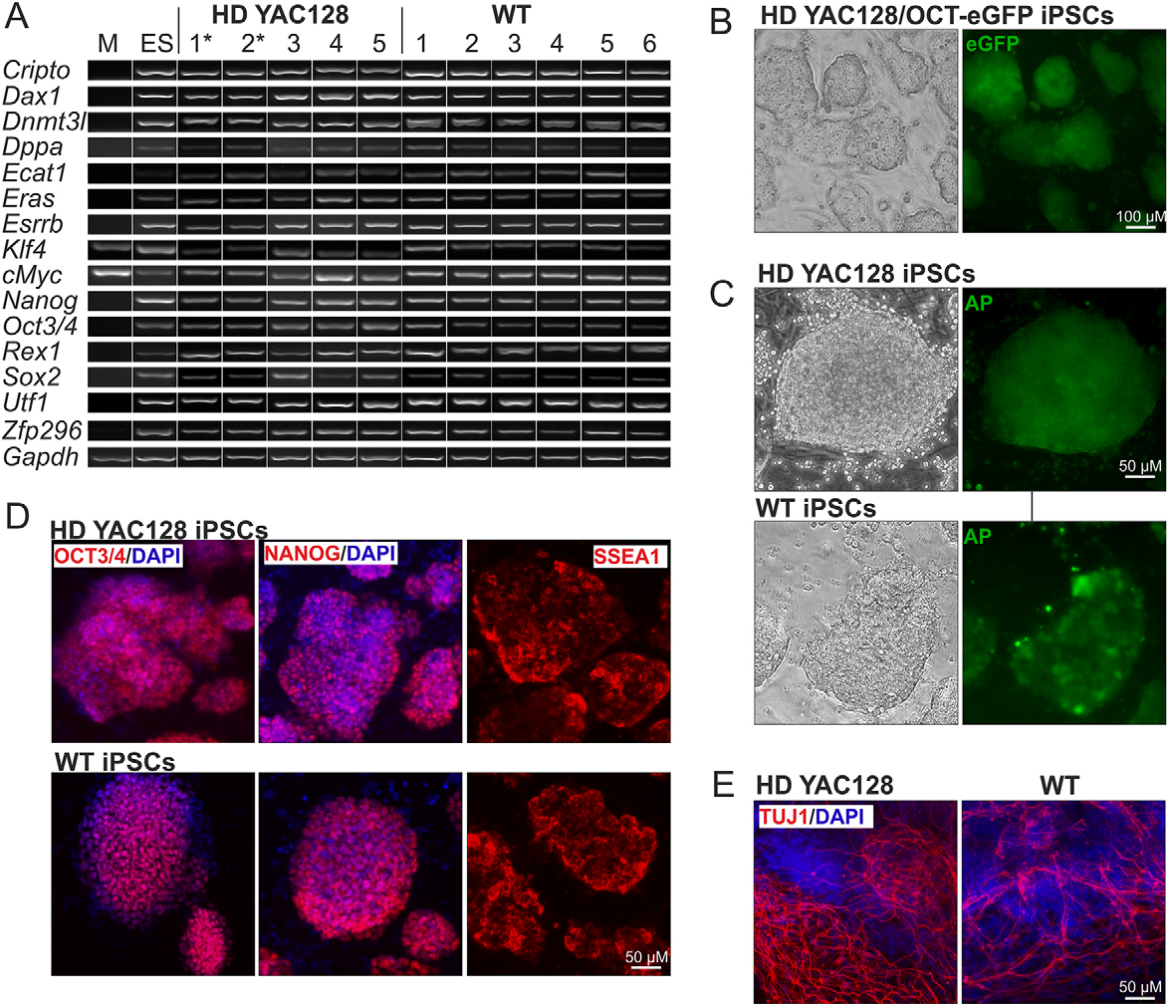
**Generation of iPSCs using cells isolated from the YAC128 HD model.** (A) Detection of pluripotency markers in HD YAC128 iPSCs (numbered clones), wild-type (WT) mouse iPSCs (numbered clones), a mouse embryonic stem cell line (ES) and mouse embryonic fibroblasts (M) using RT-PCR analysis. * indicates individual YAC128/Oct-eGFP cell lines. (B) The YAC128/Oct-eGFP iPSC lines showed eGFP fluorescence, indicating the expression of endogenous Oct3/4 protein. (C) Alkaline phosphatase (AP) was present in HD YAC128 and WT iPSCs. (D) The HD YAC128 and WT iPSCs demonstrated the expression of Oct3/4, Nanog and SSEA1 proteins, as determined using immunostaining. (E) Immunostaining for neuronal marker Tuj1 after 14 days of *in vitro* differentiation of HD YAC128 and WT iPSCs.

We generated HD iPSC lines by reprogramming adult skin fibroblasts isolated from YAC128 (Slow et al., 2003), YAC128/Oct-eGFP and wild-type (WT) mice. The fibroblasts were reprogrammed using a piggyBac transposon-based system (Yusa et al., 2009, 2011) that drove the expression of five reprogramming factors (OCT3/4, SOX2, KLF4, cMYC and LIN28), which we adapted for use with adult fibroblasts (supplementary material Fig. S1A). We established more than 50 lines of primary iPSCs containing the genome-integrated reprogramming cassette. For the seamless excision of the reprogramming cassette, the selected clones were electroporated with the piggyBac transposase construct and negatively selected through incubation with the thymidine-kinase substrate fialuridine (FIAU). The surviving colonies were individually harvested and confirmed to be free of the transposon through PCR genotyping (supplementary material Fig. S1B). Five HD and six WT iPSC cell lines were selected for further analysis. The morphology of the selected cell lines was indistinguishable from that of the control mouse ESCs. RT-PCR analysis of the selected cell lines revealed expression of the pluripotency markers *Sox2*, *Klf4*, *cMyc*, *Esrrb*, *Utf1*, *Nanog*, *Oct3/4*, *Cripto*, *Dnmt3l*, *Rex1*, *Ecat1*, *Eras*, *Zfp296*, *Dax1* and *Dppa* (Fig. 1A). The expression levels of the analyzed markers were comparable to those observed in mouse ESCs. Both HD and WT iPSCs exhibited similar expression levels of OCT3/4 mRNA and protein (supplementary material Fig. S2A,B), and the iPSC lines generated from YAC128/Oct-eGFP cells showed green fluorescence corresponding to Oct-eGFP expression (Fig. 1B). The HD and WT iPSCs also showed similar expression levels of other pluripotency genes (*Dax1*, *Dnmt3l* and *Rex1*; supplementary material Fig. S2A) and of alkaline phosphatase activity, which is another hallmark of pluripotency (Fig. 1C). The HD and WT iPSC colonies both exhibited intense nuclear immunostaining for OCT3/4 and NANOG, and cell-surface immunostaining for SSEA1 (Fig. 1D). To analyze their differentiation potentials, the HD and WT iPSCs were tested using *in vitro* and *in vivo* differentiation assays. Following the formation of embryoid bodies, the cells readily differentiated into neuronal lineage cells, showing long neuronal processes that were positive for TUJ1 expression (Fig. 1E). Cells of lineages originating from the other germ layers were also present, e.g. beating cardiomyocytes (data not shown). Upon injection into SCID and FVB/J mice, our iPSCs formed teratomas in which we detected tissues originating from all three germ layers (supplementary material Fig. S3).

### Neuronal differentiation of YAC128 iPSCs preferentially increased the level of mutant huntingtin protein

**Fig. 2.**
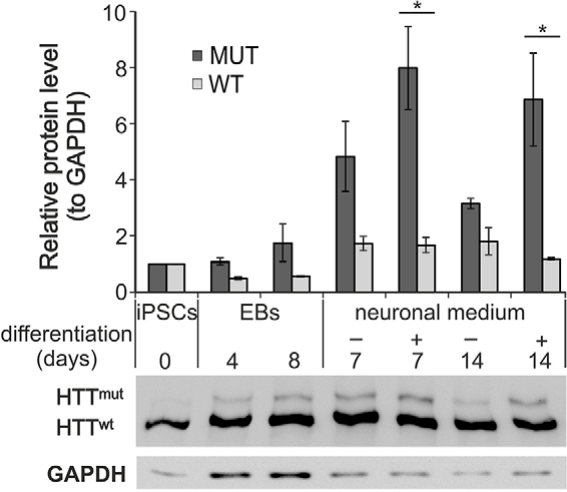
**The expression of mutant huntingtin increased upon the differentiation of the YAC128 iPSCs.** Protein lysates for western blotting analysis were prepared on days 4 and 8 of the formation of non-adherent embryoid bodies (EB4 and EB8 lanes, respectively), on days 7 and 14 of adherent differentiation (after 4 days of EB formation) in either FBS-containing medium (−) or N2B27 neuronal medium (+). The relative level of expression of mutant huntingtin increased several fold during adherent differentiation, whereas the increase in mouse wild-type (WT) huntingtin was not significant. The level of increase in mutant huntingtin was significant under neuronal-cell culture conditions. *P<0.01 in Bonferroni post-test.

The YAC128 and YAC128/Oct-eGFP iPSC lines contained a human *HTT* transgene (supplementary material Fig. S4A) and expressed both mutant human huntingtin mRNA (supplementary material Fig. S4B) and protein (supplementary material Fig. S4C). In immunoblots, the mutant huntingtin protein was represented by bands of noticeably lower intensity compared with those of mouse huntingtin. Similarly, bands of mutant huntingtin of lower intensity than that of the bands of normal huntingtin were observed in samples of human 4281 HD fibroblasts. We investigated the levels of the expression of the mutant and normal huntingtin proteins upon iPSC differentiation. We found that, in cells that differentiated toward neurons, the level of mutant huntingtin was increased several fold, whereas the degree of increase in mouse WT huntingtin was not significant (Fig. 2; time of differentiation×allele interaction, *P*=0.0192; main effect of time, *P*=0.0002; two-way ANOVA; Bonferroni post-test, *P<0.01). Our results indicated that mutant huntingtin had accumulated in neuronal cells at an early stage of differentiation.

### YAC128 iPSCs showed a decrease in MAPK-ERK pathway signaling

**Fig. 3.**
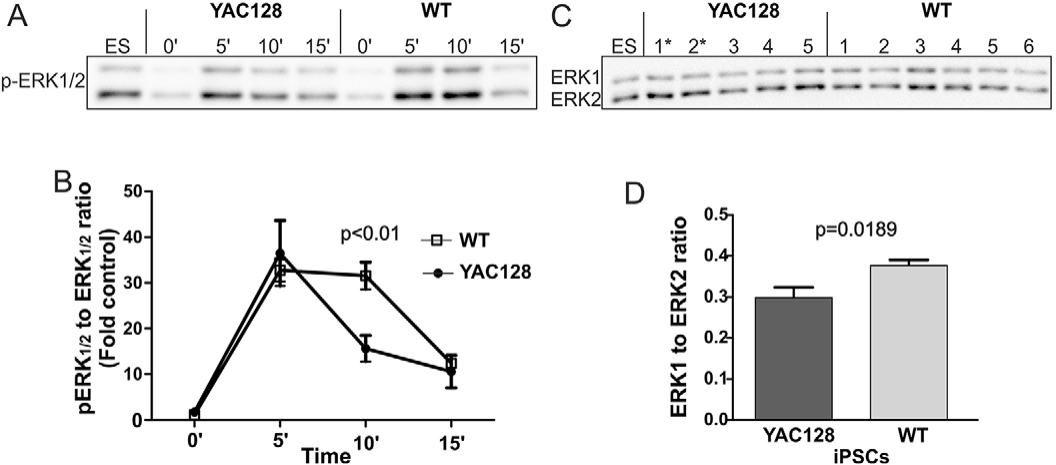
**The MAPK pathway is dysregulated in the YAC128 iPSCs.** (A) Representative time-course of ERK1/2 phosphorylation stimulated by bFGF in the YAC128 and wild-type (WT) iPSC lines. The ES lane represents a standard protein lysate that was used to normalize the band intensity among experiments. (B) Diagram demonstrating the profile of ERK1/2 activation (levels of pERK1/2). The data for the diagram were collected by investigating the level of pERK1/2 in several different clonal lines of HD YAC128 (*n*=5) and WT iPSCs (*n*=6). ERK1/2 activation was dramatically decreased in the HD YAC128 iPSCs 10 min after stimulation using bFGF, whereas ERK1/2 activation in the WT iPSCs remained high. Two-way ANOVA; time×genotype interaction, *P*=0.0013; Bonferroni post-test. (C) Western blot analysis of the levels of total ERK1/2 proteins. (D) The analysis revealed a decreased level of total ERK1 in the YAC128 lines (*t*-test, *P*=0.0189). * indicates the YAC128/Oct-eGFP lines.

The ERK pathway has been implicated in HD pathogenesis (Bowles and Jones, 2014), and upregulation of its activity might be beneficial in mouse HD models (Maher et al., 2011). Therefore, we investigated the profile of ERK activation in YAC128 iPSCs expressing human mutant huntingtin and investigated whether HD cells in an ESC-like state exhibited dysregulation of the ERK pathway. We stimulated both YAC128 and WT iPSCs using bFGF for 5, 10 or 15 min and compared their levels of ERK pathway activation (Fig. 3A). We observed different profiles of induction of this pathway for WT and HD YAC128 iPSCs (time×genotype interaction, *P*=0.0013; main effect of time, *P*<0.0001; two-way ANOVA). In WT iPSCs, we observed a maximum of approximately a 29-fold increase in the ERK1/2 phosphorylation at 5 min compared with basal levels, whereas the level of activation observed at 15 min after stimulation had decreased to 11-fold above basal levels. In YAC128 iPSCs, we observed a similar peak of ERK1/2 activation at 5 min after stimulation. However, the activation of ERK1/2 recorded at 10 min after stimulation had decreased 2.3-fold, whereas the level of ERK1/2 activation in WT iPSCs remained unchanged (*P*<0.01, Bonferroni post-test; Fig. 3B). In addition, we found that the level of basal activation of ERK1/2 in YAC128 iPSCs was 1.56-fold higher than that in WT iPSCs, although this difference was not significant (*P*=0.08). Moreover, the analyses of the levels of the total ERK1 and 2 proteins revealed that the total ERK1 protein levels were significantly lower in the YAC128-iPSC cells (Fig. 3C,D).

### HD YAC128 iPSCs showed increased phosphorylation of β-catenin

**Fig. 4.**
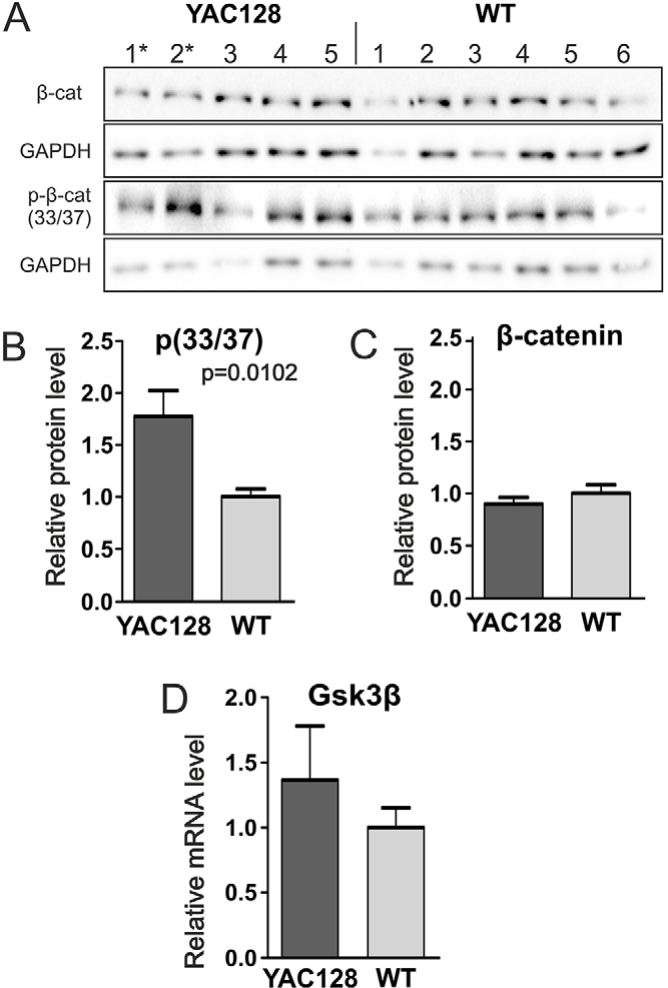
**The level of β-catenin phosphorylation was increased in YAC128 iPSCs.** (A) The levels of total β-catenin, p-β-catenin (S33/37) and GAPDH were investigated in clonal lines of HD YAC128 (*n*=5) and wild-type (WT) iPSCs (*n*=6) using western blotting. (B) Quantification of the level of p-β-catenin (S33/37) showed increased phosphorylation in the HD YAC128 iPSC lines without changes in (C) the level of total β-catenin protein. (D) qPCR analysis revealed a trend toward the increased expression of *Gsk3β*, but the extent of the increase was not significant. * indicates the YAC128/Oct-eGFP lines.

The Wnt pathway is another transduction cascade that has been implicated in the pathogenesis of HD (Godin et al., 2010); therefore, we questioned whether YAC128 iPSCs also showed dysregulation of the Wnt pathway. We monitored the phosphorylation of β-catenin at serines 33/37, 41/45 and 675 using phospho-specific antibodies. Western blotting analysis revealed an increase in the level of β-catenin phosphorylation at serines 33/37 in YAC128 iPSCs versus WT iPSCs (Fig. 4A,B), whereas the levels of serines 41/45 and 675 phosphorylation were similar in the YAC128 and WT iPSCs (supplementary material Fig. S5). However, there was no difference in levels of total β-catenin protein in these cells. Interestingly, a trend toward an increased expression of *Gsk3β* mRNA in the YAC128 iPSCs was observed (Fig. 4C); however, this change was not significant.

### The YAC128 iPSCs demonstrated increased SOD1 expression and the presence of other SOD1-immunoreactive compounds

**Fig. 5.**
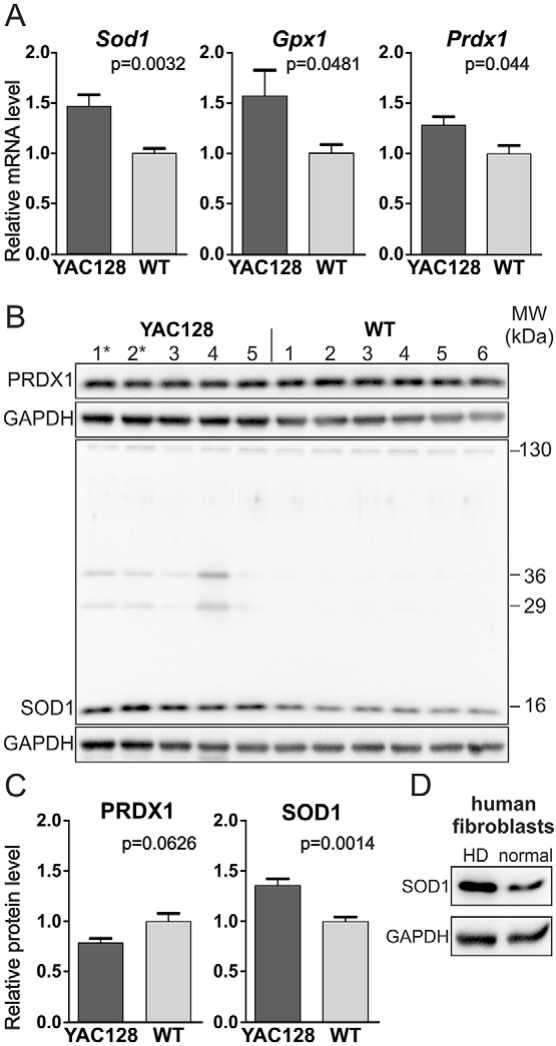
**SOD1 was overexpressed in the YAC128 iPSCs.** (A) Real-time qPCR analysis of the expression of oxidative-stress-related genes revealed the increased expression of *Sod1* and less significant differences in the levels of *Gpx1* and *Prdx1* mRNAs. (B,C) Western blotting analyses of several clonal lines revealed the increased expression of SOD1 protein but not PRDX1 protein in HD YAC128 iPSCs. (B) Notably, additional SOD1-immunoreactive bands of approximately 29 and 36 kDa were observed in HD YAC128 iPSCs but not in WT iPSCs. * indicates the YAC128/Oct-eGFP lines. (D) Increased expression of SOD1 protein was also observed in the human HD fibroblast line GM04281 (Coriell) using western blotting.

HD is associated with the increased production of free radicals and the resultant oxidative stress, which leads to neuronal dysfunction (Hands et al., 2011; Valencia et al., 2013). Antioxidant-related proteins defend cells from oxidative stress and free radicals. To determine whether the expression of such proteins was induced in early undifferentiated HD YAC128 iPSCs, we investigated the expression levels of *Sod1*, *Gpx1* and *Prdx1*. The *Sod1* mRNA expression level was increased in the HD YAC128 iPSCs, and the differences found in the expression levels of *Gpx1* and *Prdx1* genes were less statistically significant (Fig. 5A). Subsequently, we analyzed SOD1 protein expression levels and found that expression of its 16-kDa monomeric form was also increased in the YAC128 iPSCs (Fig. 5B,C). A similar increase in the level of SOD1 expression was observed in the human HD fibroblast cell line (GM04281, Fig. 5D). Interestingly, other SOD1-immunoreactive bands were also selectively present in the protein lysates of the YAC128 iPSCs. We detected SOD1-immunoreactive compounds with apparent molecular weights of approximately 29 and 36 kDa (Fig. 5B). These compounds might represent SOD1 oligomers, and they were virtually undetectable in the WT iPSCs.

### Human HD iPSCs established from HD and juvenile HD patients showed dysregulation of HD markers

**Fig. 6.**
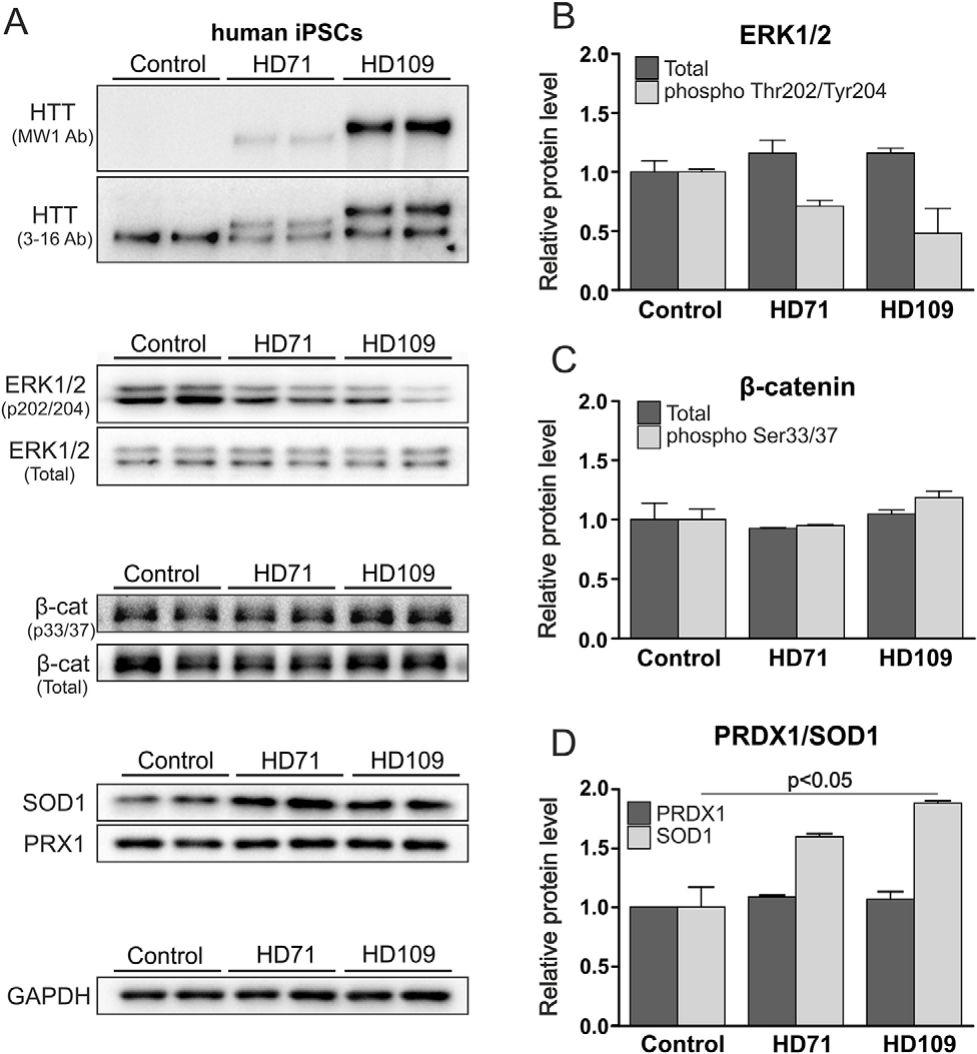
**Human HD iPSCs show dysregulation of HD markers.** (A) HD 109 CAG and HD 71 CAG cell lines contained both normal and mutant huntingtin. Western blots demonstrate decreased ERK1/2 phosphorylation, increased SOD1 expression, and unchanged PRDX1 and β-catenin protein levels. Ctrl, control. (B) A twofold decrease in the phosphorylation of ERK1/2 in the juvenile HD 109 CAG line and a milder 1.4-fold decrease in the HD 71 CAG line were detected after quantification. (C) The levels of total and 33/37 phosphorylated β-catenin remained unchanged. (D) SOD1 expression was dysregulated in human HD iPSCs, showing an increase of 1.88- and 1.6-fold in the juvenile HD 109 CAG cells and HD 71 CAG lines, respectively (1-way ANOVA *P*=0.0182; *P*<0.05, Bonferroni post-test).

We next tested whether the phenotypes observed in mouse iPSCs could also be detected in human HD patient-derived iPSCs. Several HD iPSC lines were acquired from the NINDS repository. Two lines contained 71 CAG repeats in *HTT* and originated from a 20-year-old HD patient, and another two lines contained 109 CAG repeats and originated from a 9-year-old juvenile HD patient. All HD lines contained both normal and mutant huntingtin, whereas the control lines contained only normal huntingtin (Fig. 6A). The total protein was isolated from human iPSCs, and the expression levels of phosphorylated and total ERK1/2, phosphorylated and total β-catenin, and SOD1 were examined by immunoblotting (Fig. 6A). In our studies (two lines of each genotype; three technical replicates), we observed a twofold decrease in the phosphorylation of ERK1/2 in the juvenile HD 109 CAG line and a milder 1.4-fold decrease in the HD 71 CAG line when compared with control lines (Fig. 6B). Notably, human pluripotent stem cells are dependent on an active ERK1/2 pathway and Essential 8 medium contains bFGF. Therefore, relatively high levels of ERK1/2 phosphorylation were observed in western blots without performing additional bFGF stimulation, which was inevitable for ERK assay in mouse iPSCs. Additionally, the SOD1 expression was dysregulated in human HD iPSCs, showing an increase of 1.88- and 1.6-fold in juvenile HD 109 CAG lines and HD 71 CAG lines, respectively (Fig. 6D). This increase in SOD1 expression reached statistical significance (1-way ANOVA, *P*=0.0182; *P*<0.05, Bonferroni post-test). The levels of PRDX1 protein (Fig. 6D) and β-catenin (Fig. 6C) remained unchanged. In conclusion, the protein expression patterns observed in human HD and juvenile-HD iPSCs were similar to the changes detected in YAC128 iPSCs.

### The p53 pathway was dysregulated in human HD iPSCs and YAC128 iPSCs

**Fig. 7.**
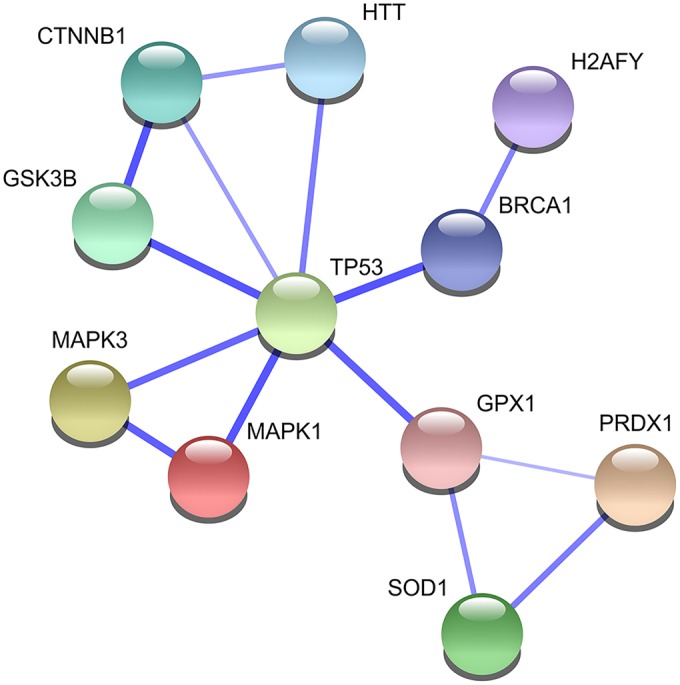
**Molecules participating in the pathways that were dysregulated in the HD YAC128 iPSCs interact with p53 protein.** The graph was generated using data obtained from the STRING 9.1 database.

**Fig. 8.**
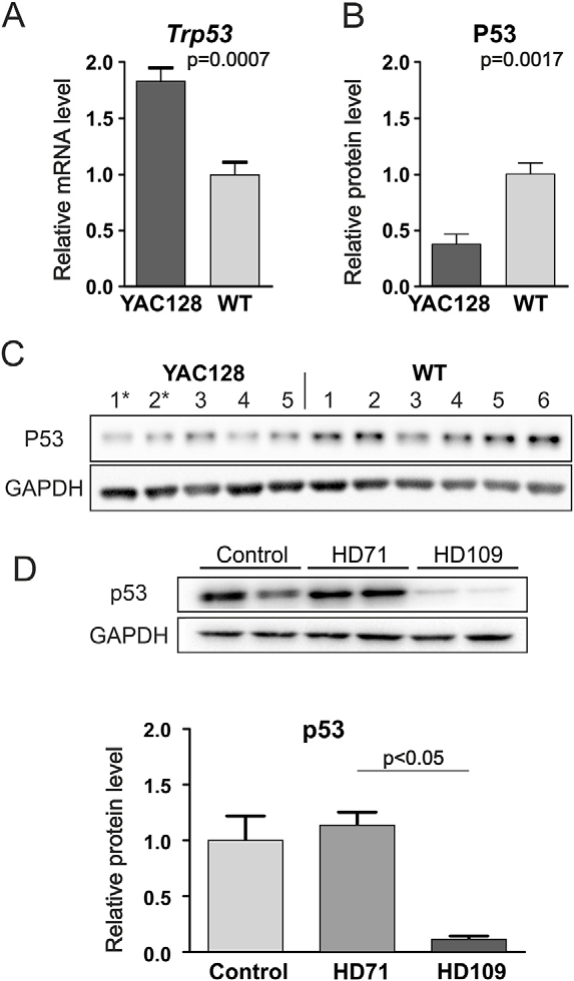
**The levels of p53 (*Trp53*) mRNA and protein were altered in HD iPSCs.** (A) Real-time qPCR analysis revealed the increased expression of *p53* mRNA in HD YAC128 iPSCs. (B,C) Western blotting and densitometric analysis revealed that p53 protein levels were decreased in the HD YAC128 iPSCs. * indicates the YAC128/Oct-eGFP lines. (D) p53 protein levels were decreased in human juvenile HD iPSC lines but not in human HD 71 iPSC lines (*P*=0.0285, 1-way ANOVA; *P*<0.05, Bonferroni post-test).

In our search for molecular pathways that were dysregulated in the YAC128 iPSCs, we detected altered activation profiles of the ERK and Wnt pathways and the accumulation of mutant huntingtin and SOD1. p53 is a pleiotropic molecule that binds huntingtin (Steffan et al., 2000), interacts with ERK and Wnt pathways and plays a role in oxidative stress. Data mining using the STRING 9.1 database (Franceschini et al., 2013) revealed that all molecules with dysregulated processes in HD YAC128 iPSCs interact with p53 (Fig. 7). In our studies, investigation of the level of p53 expression revealed the induction of the expression of *Trp53* mRNA (Fig. 8A); however, the level of the protein was strongly reduced in the HD YAC128 iPSCs (Fig. 8B,C). Interestingly, in human iPSCs, we observed a strong (tenfold) decrease in p53 protein in the juvenile HD 109 CAG line (Fig. 8D). However, this effect was absent in the HD 71 CAG line (*P*=0.0285, 1-way ANOVA; *P*<0.05, Bonferroni post-test).

### Expression of other genes in HD YAC128 iPSC

The H2A histone family, member Y (*H2afy*) gene, which encodes the macroH2a1 histone variant, has been found to be a clinically relevant HD biomarker that is overexpressed in the blood (mRNA) and brains (protein) of HD patients, as well as in the brains of two HD mouse models (Hu et al., 2011). Consistent with these results, we detected the upregulated expression of *H2afy* mRNA in YAC128 iPSCs (supplementary material Fig. S6A); however, this upregulation was not accompanied by an increased level of macroH2A1 protein (supplementary material Fig. S6B).

We found no differences in the levels of mRNA expression (supplementary material Fig. S7) for genes involved in cholesterol biosynthesis (*Dhcr7a*) and the TGF-β (*Lefty1/2*) pathway, which are dysregulated in HD R6/2 iPSCs (Castiglioni et al., 2012) and in HD knock-in ESCs (Jacobsen et al., 2011), respectively.

## DISCUSSION

We investigated HD molecular markers using both HD YAC128 and human HD iPSCs. YAC128 and other HD mouse models faithfully recapitulate the pattern of molecular changes observed in human postmortem caudate tissue from individuals with HD using high-throughput mRNA expression profiling (Hodges et al., 2006; Kuhn et al., 2007). The HD phenotype in the YAC128 mouse model progresses slowly and corresponds relatively well to the human condition. However, there are reports that suggest that the YAC128 phenotype is similar to juvenile HD (Brooks et al., 2012). Interestingly, its 128 CAG repeats originate from a juvenile HD patient (Slow et al., 2003). Overall, the YAC128 model possesses relatively high construct and face validity, making it a good candidate for the generation of iPSCs. An important advantage of using mouse iPSCs is their defined genetic background, originating from somatic cells of donor mice. In this respect, individual human iPSC lines bring greater genetic variability, which might affect the discovery of HD-related phenotypes. In addition, mouse iPSCs exist in a more primitive ‘naïve’ pluripotent state compared with the more primed state of human iPSCs (Rossant, 2015). Taken together, both HD iPSC models are suitable and complementary for determining early HD phenotypes.

YAC128 and WT iPSCs with excised reprogramming cassettes expressed the mRNA and protein of key endogenous pluripotency markers, including OCT3/4 and NANOG. In addition, the iPSCs were able to differentiate into cells of lineages that represented all germ layers. Most importantly, the YAC128 iPSCs possessed a neuronal differentiation potential and expressed mutant huntingtin. The accumulation of mutant huntingtin is the major event in HD (Chan et al., 2010) and, upon the neuronal differentiation of the YAC128 iPSCs, the expression of mutant huntingtin increased at a substantially greater rate than did the expression of mouse huntingtin. This result might indicate the decreased turnover of the mutant protein, leading to its accumulation already during the early stage of neuronal differentiation. It is likely that the level of mutant huntingtin might also be affected by differences in the proliferative characteristics of iPSCs and iPSC-derived cells that were differentiating into neurons.

Mouse HD YAC128 iPSCs and human HD iPSCs exhibited divergent basal ERK phosphorylation levels because of different pluripotency cell stages. In mouse HD YAC128 iPSCs, the unstimulated level of ERK phosphorylation was almost undetectable, whereas the level observed in human iPSCs was the result of high ERK and bFGF signaling. Regardless of these differences, both HD YAC128 iPSCs and human HD iPSCs showed decreased levels of ERK phosphorylation, exhibiting altered profiles of ERK induction minutes after stimulation (HD YAC128) and decreased levels of ERK phosphorylation under conditions of constant activation of the pathway (human HD iPSC). Symptoms of early and late HD have been demonstrated in the YAC128 HD mouse model, including early susceptibility and later resistance to excitotoxicity induced by quinolinic acid as well as early hyperactivity and later decreases in activity (Slow et al., 2003). Mutant huntingtin is known to affect the activation of the MAPK signaling pathway (Bodai and Marsh, 2012), and the dysregulation pattern is biphasic, depending on the stage of HD. Young YAC128 mice show decreased phosphorylation of ERK, whereas 1-year-old YAC128 mice show an increased level of p-ERK (Gladding et al., 2014). Differences in ERK phosphorylation are also observed in the R6/2 HD mouse model, in which an increased level of phosphorylation is apparent during the later stages of the disease (Saavedra et al., 2011). Therefore, the decreased level of p-ERK in iPSCs demonstrates that the initial molecular changes associated with HD are present early on, when the cells are in the pluripotent stage.

The decrease in the level of ERK1/2 phosphorylation in the HD iPSCs could be explained by several mechanisms. Because huntingtin is responsible for the vesicular trafficking of receptors (Caviston and Holzbaur, 2009; Liot et al., 2013), a plausible explanation might be that the trafficking of fibroblast growth factor receptors (FGFRs) was altered. Other conditions that might affect ERK1/2 signaling are the recently reported enhancement of the expression of protein tyrosine phosphatase (Saavedra et al., 2011) or the activation of proteases, such as calpain (Gladding et al., 2014). Interestingly, conditions that activated ERK signaling improved the HD phenotype of model mice (Maher et al., 2011). Collectively, the data indicated that enhancing ERK1/2 signaling in non-symptomatic HD might be a valid therapeutic strategy.

Several authors have suggested that there is crosstalk between the ERK1/2 and Wnt pathways (Bikkavilli and Malbon, 2009; Ji et al., 2009). In addition, dysregulation of the Wnt pathway and the accumulation of phosphorylated forms of β-catenin both interfere with polyglutamine toxicity in cellular and *in vivo* models of HD (Carmichael et al., 2002; Dupont et al., 2012; Godin et al., 2010). Therefore, we investigated whether canonical Wnt signaling was dysregulated in HD YAC128 iPSCs and human HD iPSCs. We found that unstimulated YAC128 iPSCs maintained a higher level of β-catenin phosphorylation at positions 33/37 than WT iPSCs. Human juvenile HD 109 CAG iPSCs and HD 71 CAG iPSCs did not show changes in β-catenin phosphorylation. The differences between mouse and human iPSCs might be due to differences in the pluripotent state or in the polyglutamine (polyQ) content of huntingtin proteins.

Residue 33/37 of β-catenin is phosphorylated by GSK3β, and we detected a non-significant increase in the level of *Gsk3β* mRNA expression in HD YAC128 iPSCs. In addition, young presymptomatic HD animals accumulate GSK3β and huntingtin in lipid rafts, and inhibiting GSK3β expression increased the survival rate of HD neurons (Carmichael et al., 2002; Valencia et al., 2010). Moreover, it was demonstrated that β-catenin directly binds to FOXO to enhance FOXO transcriptional activity in mammalian cells (Essers et al., 2005). Integration of canonical β-catenin signaling, sirtuin and FOXO signaling protects against mutant huntingtin toxicity (Parker et al., 2012). In turn, FOXO neuroprotective activity is repressed by induction of Ryk expression in HD neurons (Tourette et al., 2014). Together, a decreased level of canonical Wnt activation might represent a promising marker of early HD symptoms, and restoration of dysregulated β-catenin and FOXO transcriptional activity might serve as a possible therapeutic strategy in HD. In addition, increased β-catenin phosphorylation in HD pluripotent cells might influence several future stages of neuronal differentiation. For instance, inhibition of Wnt–β-catenin signaling with Dkk1 during the mid and late stages of cortical neurogenesis suppresses neuronal production (Munji et al., 2011), whereas, in adult mice, it triggers degeneration of striatal synapses and impairs motor coordination (Galli et al., 2014).

A study of symptomatic R6/1 animals demonstrated that they had increased levels of inhibitory phosphorylation of GSK3β, suggesting the decreased activity of this molecule in the HD R6/1 model (Lim et al., 2014). Therefore, it cannot be excluded that signaling dependent on GSK3β can change, depending on the stage of HD.

In addition, the 41/45 serines of β-catenin are generally phosphorylated before serines 33/37, and we detected the phosphorylation of β-catenin at serines 41/45 and 675. The phosphorylation levels of serines 41/45 and 675 were relatively high and were similar in the WT and HD YAC128 iPSCs, suggesting similar activities of CK1α and PKA in our iPSCs. Normal somatic brain cells generally demonstrate low levels of phosphorylated β-catenin (Godin et al., 2010).

Oxidative stress is a molecular hallmark of HD (Ribeiro et al., 2012), and HD brain tissue shows greatly increased expression of multiple genes involved in oxidative-stress-related pathways (Sorolla et al., 2008). We also detected changes in YAC128 iPSCs regarding the expression of *Sod1*, *Prdx1* and *Gpx1*, which are genes involved in protection against oxidative stress. SOD1 expression was also increased in human HD fibroblasts and HD iPSCs. The additional SOD1-immunoreactive bands observed in mouse iPSC might represent complexes or aggregated forms of SOD1, alone or with other proteins. Interestingly, human iPSCs derived from an HD patient with 72 CAG repeats showed decreased SOD1 expression (Chae et al., 2012).

The above-mentioned dysregulation of processes involving *Mapk1* (ERK2), *Mapk3* (ERK1), *Gsk3β*, *Ctnnb1* (β-catenin), *Gpx1*, *Prdx1* and *Sod1*, and the presence of mutant HTT, seem to build a network of molecular interactions. The STRING database predicted p53 as a molecule that interacts with all of the above-identified pathways and molecules. p53 protein levels were lower in HD YAC128 iPSCs than in WT iPSCs, but the level of *Trp53* mRNA was higher in HD YAC128 iPSCs, which might be the result of increased degradation and/or the disturbed production of p53 protein in HD YAC128 iPSCs. The decreased p53 protein expression was also observed in human juvenile HD 109 CAG cells but was unchanged in human HD 71 CAG cells. Interestingly, the observed decrease in p53 protein was not detected in NSCs or differentiated HD cells, where p53 expression is typically increased and is associated with apoptotic processes (Bae et al., 2005; Ehrnhoefer et al., 2014; The HD iPSC Consortium, 2012). Summarizing, the observed dysregulation of p53 expression in HD iPSCs supports the involvement of the p53 pathway in the early stages of HD pathogenesis. In addition, further research including functional assays is needed to elucidate developmental effects of early HD changes.

The identification of very early molecular signs of HD could enhance our understanding of neurodegenerative diseases that are typically regarded as age-related processes. In particular, our findings will facilitate more precise identification of the onset of HD and will have implications for therapeutic approaches, including cellular therapy using autologous iPSCs. Moreover, our study identified early markers that can be investigated in iPSCs to validate the efficacy of drugs in cell culture as well as *in vivo*.

## MATERIALS AND METHODS

### HD mice and derivation of fibroblasts

YAC128 HD mice and WT littermates were obtained from The Jackson Laboratory (Bar Harbor, ME, USA; JAX Mice #004938, FVBN/J background). Additionally, for the assessment of fibroblast reprogramming, we generated a crossbreed of YAC128 and Oct-eGFP mice (Lengner et al., 2007) (JAX Mice #008214, B6;129S4/SvJae background). The cultured mouse adult fibroblasts were derived from back-skin biopsies of 8- to 10-week-old mice. Briefly, the skin fibroblasts were cultured using the explant technique in HEPES-buffered DMEM (Sigma-Aldrich, St Louis, MO, USA) supplemented with 10% FBS originating from New Zealand (Sigma-Aldrich), 2 mM L-Gln (Sigma-Aldrich), 1× antibiotic-antimycotic mixture (Sigma-Aldrich) and 10 μg/ml L-ascorbic acid (Sigma-Aldrich). Human HD fibroblasts, line GM04281, were obtained from Coriell (Coriell Cell Repository, Camden, NJ, USA). The protocols for the maintenance and handling of the animals were approved and monitored by the Local Ethical Commission for Animal Experiments in Poznan.

### Generation and culturing of the mouse iPSC lines

The HD YAC128 and WT iPSCs were generated using a five-factor (Oct3/4, Sox2, Klf4, cMyc and Lin28) piggyBac transposon-based system (Yusa et al., 2009, 2011) optimized for mouse adult fibroblasts. We also established iPSCs from YAC128/Oct-eGFP crossbred mice to monitor the expression of endogenous OCT3/4 during reprogramming, culturing and differentiation.

Briefly, 2×10^6^ detached fibroblasts at passages 2-4 were electroporated (BTX, ECM 830) with 6 μg of pPB-CAG.OSKML-puΔtk and 4 μg of the hyPBase plasmids in DMEM-HEPES cell suspension. Subsequently, cells were seeded onto 60-mm dishes (1×10^6^ fibroblasts/dish) coated with mitomycin-C-inactivated MEF feeder cells in fibroblast medium. The medium was changed the next day (day 1). The medium used later, during reprogramming, consisted of Knockout DMEM (Life Technologies, Carlsbad, CA, USA), 2 mM L-Gln, 1× antibiotic-antimycotic mixture, 1× MEM non-essential amino acids (Sigma-Aldrich), 1000 U/ml LIF (ORF Genetics, Kopavogur, Iceland), 0.1 mM β-mercaptoethanol (Sigma-Aldrich) and 10 μg/ml L-ascorbic acid (Sigma-Aldrich) containing either 15% FBS (F15 medium) or 15% KnockOut Serum Replacement (Life Technologies; K15 medium). From day 2 to 8, the cells were grown on F15 medium supplemented with 2 mM valproic acid (VPA, Sigma-Aldrich), whereas, from day 9, F15:K15 (1:1 mix) medium lacking VPA was used. The medium was changed every 2 days. The medium used during reprogramming was supplemented with L-ascorbic acid, which was removed after the initial selection of clones. Individual iPSC clones were harvested between days 15 and 28 and were expanded in K15 medium without L-ascorbic acid, with daily medium renewal. The cells were dissociated using the TrypLE Select reagent (Life Technologies). To excise the reprogramming cassette, the iPSC clones (1×10^6^ cells; passages 2-4) were electroporated with 4 μg of hyPBase and were seeded on a feeder layer. At day 3, the iPSCs were passaged and, from day 4, they were grown for 5 days under selection conditions using 0.2 μM FIAU (Moravek Biochemicals, Brea, CA, USA). After another 5 days of growth without the selection reagent, the emerging colonies were selected, expanded and genotyped to confirm the excision of the transposon. The ESCs used in the study were Gibco (C57BL/6) mouse ESCs (Life Technologies), and the TrypLE Select reagent (Life Technologies) was used to dissociate the pluripotent stem cells for passaging.

### Human HD iPSCs

Human episomal HD and control iPSC lines were obtained from the NINDS Human Genetics Resource Center DNA and Cell Line Repository (https://catalog.coriell.org/1/ninds). For the analysis, we used two clonal HD lines with 71 CAG repeats (ND42228, ND42230; derived from a 20-year-old patient), two juvenile HD clonal lines with 109 CAG repeats (ND42223, ND42224; derived from a 9-year-old patient) and two control lines with 17/18 (ND41658) and 21 (ND42245) CAG repeats. Human iPSCs were cultured in chemically defined conditions in Essential 8 medium (Life Technologies) and grown on recombinant human vitronectin-coated surfaces (VTN-N, Life Technologies). Cells were passaged using gentle dissociation with 0.5 mM EDTA in PBS. Total protein was isolated after three to five passages after thawing of the NINDS samples.

### Genotyping, RT-PCR and real-time qPCR

For genotyping, DNA was isolated using a Spin Column Genomic DNA Kit (Bio Basic Inc., Markham, Canada). Genotyping to determine the excision of the transposon was performed using multiplex PCR using a set of primers specific for the *Tcrd* gene (the internal wild-type control) and either pPB-OTS or pPB-KM2 primers specific for the OCT/T2A/SOX or KLF/T2A/cMYC linker in the reprogramming cassette, respectively. A list of the primers used in this study is provided in supplementary material Table S1. Genotyping to determine the presence of the YAC128 transgene was performed using a pair of primers specific for the human intronic *HTT* sequence and a pair of primers specific for the internal wild-type control (*Tcrd*). All genotyping analyses were performed using Touchdown PCR with the following cycling conditions: 3 min at 94°C; 12× [35 s at 94°C, 45 s at 64°C (–0.5°C/cycle) and 45 s at 72°C]; 25× (35 s at 94°C, 30 s at 58°C and 45 s at 72°C); and, finally, 2 min at 72°C. The reaction products were separated on 1.3% agarose gels in TBE buffer and were stained using ethidium bromide.

The total RNA was isolated using TriReagent (MRC, Cincinnati, OH, USA) and reverse transcription was performed using SuperScript^®^ III reverse transcriptase (Life Technologies). For the real-time qPCR, SYBR^®^ Select Master Mix (Life Technologies) was used, and the reactions were performed using a LightCycler 480 II (Roche) instrument. The data was analyzed using LightCycler 480 software (release 1.5.1.62). For each primer pair a standard curve was prepared using the Second Derivative Maximum method. The efficiencies of standard curves and Ct values for each sample are given in supplementary material Table S2. The relative expression was calculated with the E-method with Gapdh as a reference (Ref) gene. Target/Ref ratios for each sample were normalized to mean ratio of WT samples and these values were used for statistical analyses.

Both the human *HTT* and mouse *Htt* mRNAs were amplified by RT-PCR using primers that result in products spanning the CAG-repeat-containing exon 1 and the exon 2 junction. Because the primers were designed for human *HTT* and there was one non-complementary nucleotide for the mouse *Htt* in the reverse primer, it cannot be excluded that the amplification of the human and mouse huntingtin mRNA products proceeded with slightly different efficiencies. Thus, the direct comparison of the levels of the *HTT* and *Htt* mRNAs was not possible.

### Immunostaining

For immunostaining, the cells were cultured in 12-well dishes on gelatin- and feeder-cell-coated coverslips. The cells were washed using PBS, fixed by incubation with 4% PFA for 15 min at room temperature (RT), washed and permeabilized (except when immunostaining SSEA1, which is a surface marker) using 0.3% Triton X-100 in PBS for 10 min at RT. Blocking was performed in 5% normal goat serum (Jackson ImmunoResearch) in PBS for 1 h at RT, and the primary-antibody incubation was conducted overnight at 4°C in the same buffer. The primary antibodies used were as follows: anti-SSEA1 (1:500, Millipore, MAB4301); anti-OCT3/4 (1:500, Santa Cruz Biotechnology, sc-5279); anti-NANOG (1:700, Abcam, ab80892); and anti-TUJ1 (1:500, Millipore, MAB1637). After washing using PBS, the cells were incubated for 1 h at RT with a secondary antibody, ether Cy3-conjugated goat anti-mouse or anti-donkey antibodies (Jackson ImmunoResearch) at a 1:500 dilution in PBS. A 5-min incubation in DAPI (1:10,000) dissolved in water was used for counterstaining. Additionally, the primary antibodies were omitted in the negative controls. The coverslips containing the cells were mounted on slides using anti-fade glycerol/propyl gallate mounting medium. The specimens were analyzed using an Olympus IX70 fluorescence microscope and images were captured using an Olympus DP71 camera. The alkaline-phosphatase Live Stain (Life Technologies) assay was used to determine the level of alkaline phosphatase activity, according to the manufacturer's protocol.

### *In vitro* differentiation

The iPSCs or mouse ESCs were dissociated and then transferred to differentiation medium (K15 without LIF) in non-adherent six-well plates (Corning) at a density of 5.5×10^5^/well. Embryoid bodies were allowed to form until day 4, with daily changes of the medium, and then they were transferred into gelatin-coated wells containing EB medium supplemented with FBS or N2B27 instead of KSR and were cultured until day 14, with a medium change every 2 days.

### Teratoma formation

A total of 1×10^6^ cells suspended in 100 μl of 1:1 PBS/Geltrex (Life Technologies) solution were subcutaneously injected into both dorsal flanks of SCID mice. Teratomas generally formed in 4-8 weeks and were collected when they reached approximately 1 cm in diameter and then were transferred to 4% PFA and fixed for 1 week at 4°C. The tumors were then transferred to 10%, 20% and 30% sucrose, each for 3 days, frozen on a specimen holder and cut into 10-μm sections using a cryostat (Leica). The sections were stained using H&E.

### ERK activation assay

The day before the assay was performed, the medium on mouse iPSCs was exchanged for serum-free medium without LIF, and the cells were starved for 24 h. Then, without changing the medium, 20 ng/ml bFGF was added, and the cells were incubated for 5, 10 or 15 min. After each incubation period, the medium was quickly discarded and the cells were immediately lysed using a protein-lysis buffer (see next subsection for buffer formulation). The ERK phosphorylation was directly tested in human iPSCs because the Essential 8 medium contains bFGF, which is required for growth of human iPSCs. The protein lysates were subsequently used for western blotting using ERK1/2 phospho-specific antibodies.

### Western blotting

For protein isolation, the cells were washed using PBS, lysed in a protein-lysis buffer containing 60 mM Tris base, 2% SDS, 10% sucrose, 2 mM PMSF (Sigma-Aldrich) and 1× Halt Phosphatase Inhibitor Cocktail (Thermo Scientific), and homogenized. An aliquot of 20-30 μg of total protein per lane was dissolved in loading buffer containing 2-mercaptoethanol and was boiled for 5 min. The proteins were separated using SDS-PAGE (5%/10% stacking/resolving gels) and Laemmli buffer. Huntingtin was separated in 4% stacking/5% resolving gels using commercial XT Tricine running buffer (Bio-Rad), as previously described (Fiszer et al., 2013). The proteins were transferred to nitrocellulose membranes and the blots were blocked using 5% nonfat milk and 0.1% Tween 20 in TBS and were subsequently incubated overnight at 4°C with primary antibody diluted in TBS-Tween containing 5% milk or BSA. The antibodies used were purchased from Cell Signaling Technology (Leiden, The Netherlands) unless otherwise stated and were as follows: rabbit anti-β-catenin (1:1000, cat. 8480); rabbit anti-phospho-β-catenin (Ser675) (1:1000, 4176), (Thr41/Ser45) (1:1000, 9565) and (Ser33/37) (1:1000, 2009); rabbit anti-p44/42 MAPK (ERK1/2) (1:2000, 4695), rabbit anti-phospho-p44/42 MAPK (ERK1/2) (Thr202/Tyr204) (1:1000, 4370), rabbit anti-MacroH2A1 (1:1000, 8551); mouse anti-Oct3/4 (1:1000, Santa Cruz Biotechnology, Dallas, TX, USA; sc-5279); mouse anti-p53 (1:1000; 2524); mouse anti-huntingtin (1:2000, Millipore, Billerica, MA, USA; MAB2166), rabbit anti-huntingtin N-terminal, amino acids 3-16 (1:1000, Sigma-Aldrich, H7540), mouse anti-polyQ [1:1000, MW1, Developmental Studies Hybridoma Bank (DSHB), Iowa City, IA, USA; Ko et al., 2001]; and mouse anti-GAPDH (1:10,000, Millipore, MAB374). The blots were then incubated for 2 h at RT with HRP-conjugated secondary antibodies raised against rabbit or mouse antibodies (1:2000-1:20,000 dilution, Jackson ImmunoResearch, Suffolk, UK), and the labeled bands were detected using the ECL-based SuperSignal West Pico (Thermo Scientific) substrate. All analyses were performed as three independent technical replicates.

### Statistics

The two-group comparisons of the gene expression data were conducted using the unpaired Student's *t*-test. The data regarding huntingtin expression during differentiation and ERK1/2 activation were subjected to a two-way ANOVA, followed by Bonferroni post-tests. *P*-values of less than 0.05 were considered significant. Error bars on all graphs represent s.e.m.

## Supplementary Material

Supplementary Material
