# Supplementary material for: Huntington disease iPSCs show early molecular changes in intracellular signaling, the expression of oxidative stress proteins and the p53 pathway
Source: Dis Model Mech. 2015 Sep 1;8(9):1047–57. doi: 10.1242/dmm.019406 (PMC4582098; doi:10.1242/dmm.019406)
Supplement: Supplementary Material [file supp_8_9_1047__index.html]

Supplementary Material 

# Huntington disease iPSCs show early molecular changes in intracellular signaling, the expression of oxidative stress proteins and the p53 pathway

## DMM019406 Supplementary Material

- Supplementary Material
