# Supplementary material for: Huntington disease iPSCs show early molecular changes in intracellular signaling, the expression of oxidative stress proteins and the p53 pathway
Source: Dis Model Mech. 2015 Sep 1;8(9):1047–57. doi: 10.1242/dmm.019406 (PMC4582098; doi:10.1242/dmm.019406)
Supplement: Supplementary Material [file supp_019406_DMM019406supp.pdf]

# Supplementary Figures

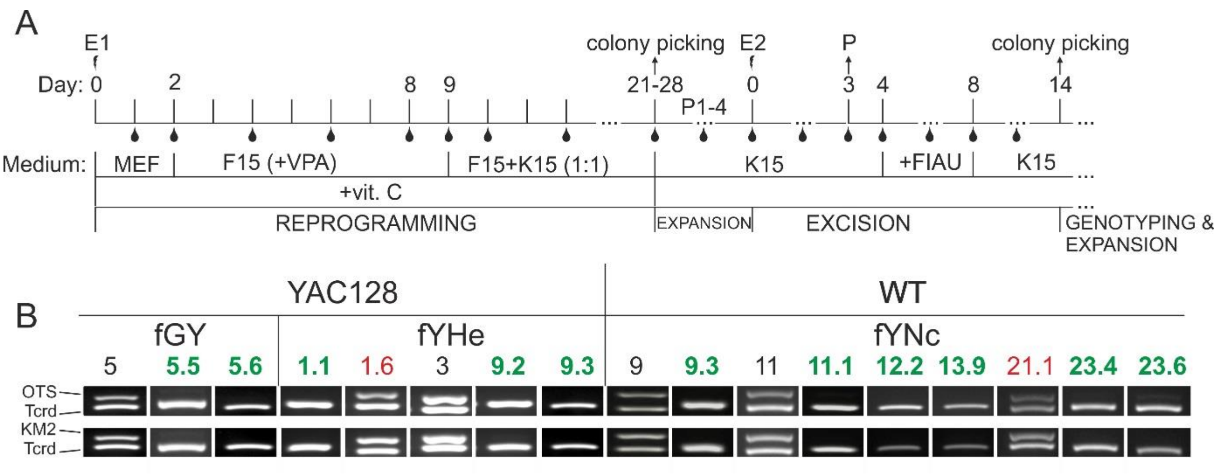


## Fig. S1

Schematic illustration of the method used for iPSC generation using the piggyBAC system. (A) Reprograming of fibroblasts into iPSCs, harvesting iPSC colonies and the excision of reprogramming cassette. The black drops, P and E indicate medium changes, passage and electroporation, respectively. (B) The excision of the cassette was validated using multiplex PCR genotyping which lack of reprogramming cassette. Oct-to-Sox (OTS) was a PCR product for which primers were designed to bind to the linker between Oct3/4 and Sox2. Similarly, KM2 was a PCR product for which the primers bound to the linker between Klf4 and cMyc. The majority of the clones exhibited the excision of the cassette after selection using FIAU and are labeled with green numbers, whereas only a few of the clones still contained the cassette (red numbers). Pre-excision clones (positive control) are labeled with black numbers. Only the clones in which excision occurred (green labeled) were used in the studies described herein.

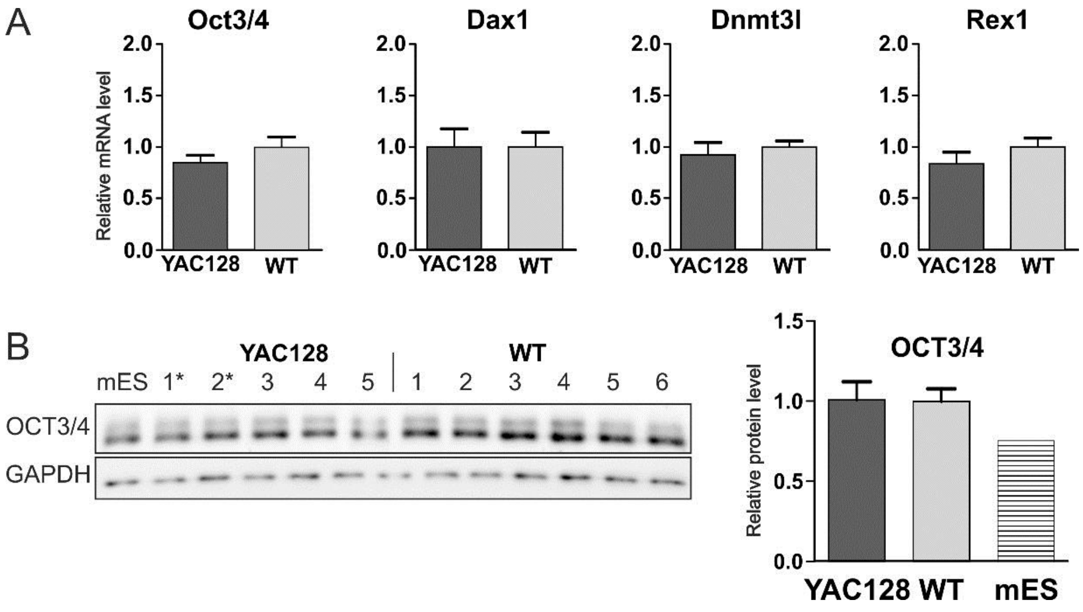


## Fig. S2

Analysis of the expression of the pluripotency genes Oct3/4, Dax1, Dnmt3l and Rex1 using (A) qPCR and (B) western blotting demonstrated no differences between the YAC128 and WT lines. *YAC128/Oct-eGFP lines.

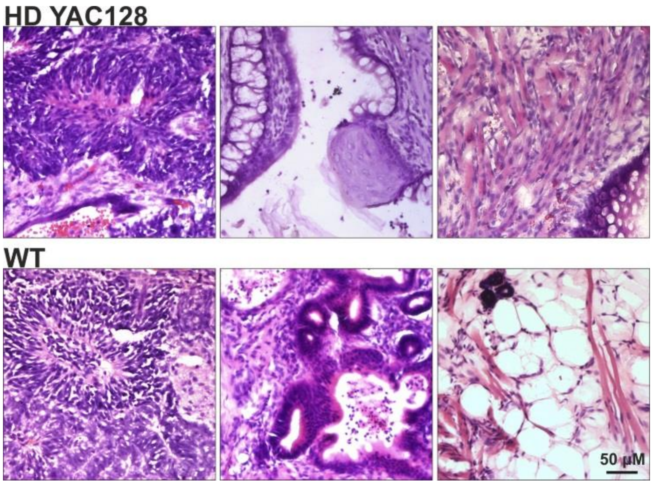


**Fig. S3**

Teratomas generated from HD-YAC128 and WT iPSCs contained cell types originating from the three germ layers: neuroepithelial cells (ectoderm); gut and respiratory epithelial cells, glands (endoderm); and muscle fibers, adipose tissue and cartilage (mesoderm).

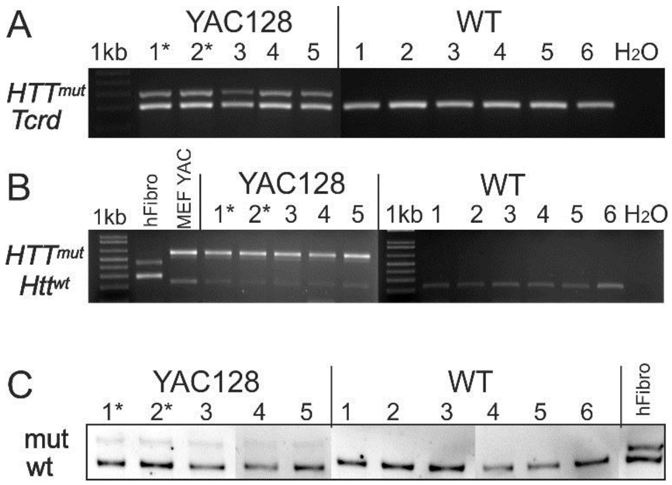


**Fig. S4**

Mutant huntingtin was expressed in YAC128 iPSCs (A) Genotyping for the presence of the mutant *HTT* gene in YAC128 and WT iPSCs. The multiplex touchdown-PCR reaction utilized endogenous *Tcrd* gene as the internal control (B) RT-PCR analysis of *HTT* mRNA expression. (C) Western blotting analysis of huntingtin expression in the HD YAC128 and WT iPSCs. (*) YAC128/Oct-eGFP lines; hFibro, human HD fibroblasts (GM04281, Coriell) containing 17 and 68 CAGs; MEF YAC, MEFs obtained from YAC128 mice.

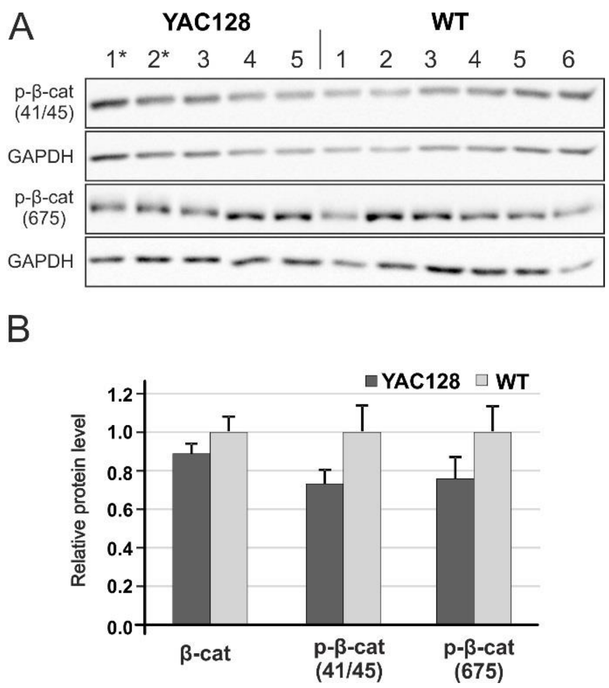


**Fig. S5**

The levels of p41/45-β-catenin and p675-β-catenin were similar in YAC128 iPSCs and WT iPSCs. (A) The levels of p41/45-β-catenin, p675-β-catenin and GAPDH were investigated in clonal lines of HD YAC128 (n=5) and WT iPSCs (n=6) using western blotting. (B) The quantification diagram for the levels of expression of p41/45-β-catenin and p675-β-catenin demonstrates similar degrees of activation in the HD YAC128 and WT iPSC lines. *YAC128/Oct-eGFP lines.

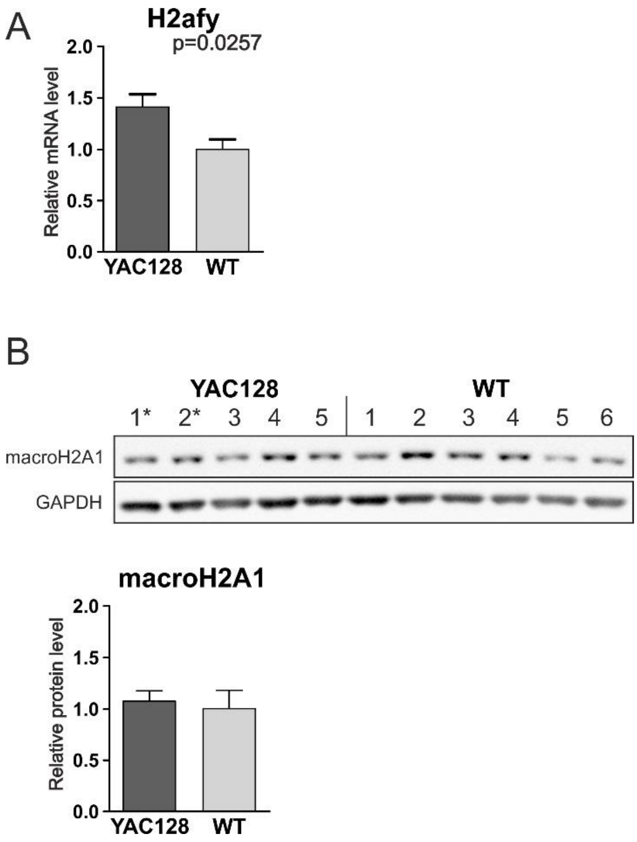


## Fig. S6

The expression of the *H2afy* gene was increased (A) in the YAC128 iPSCs. The level of the resulting histone macroH2A1 (B) protein was similar in YAC128 and WT iPSCs. *YAC128/Oct-eGFP lines.

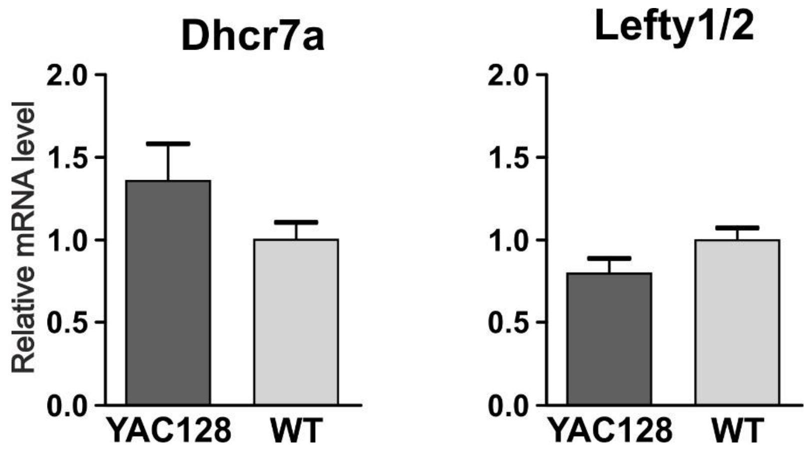


**Fig. S7**

The levels of mRNA expression of genes that play roles in lipid metabolism (*Dhcr7*), and signaling pathways (*Lefty1/2*) were similar in YAC128 and WT iPSCs, as revealed using real-time qPCR analysis.

**SUPPLEMENTARY TABLE 1. List of primers used**

| Primer name | | Sequence 5' -> 3' | Comment/Source |
|---|---|---|---|
| **Genotyping** | | | |
| pPB-OTS | F | TTCCCAACGAGAAGAGTATGAGGCTACA | transposon removal |
| | R | GCTCCGTCTCCATCATGTTATACATTGG | |
| pPB-KM2 | F | AGGCGAGAAACCTTACCACTGTGACTG | transposon removal |
| | R | AGTCGAGGTCATAGTTCCTGTTGGTGAA | |
| NI26_27 | F | CCTCTTATATATGGATGCTAATCTCATTC | YAC128 transgene |
| | R | AATACACAACACATGAGAGCATATAGAAC | |
| Tcrd | F | CAAATGTTGCTTGTCTGGTG | internal control |
| | R | GTCAGTCGAGTGCACAGTTT | |
| | | | |
| **RT-PCR** | | | |
| mCripto (Tdgf1) | F | ATGGACGCAACTGTGAACATGATGTTCGCA | (1) |
| | R | CTTTGAGGTCCTGGTCCATCACGTGACCAT | |
| mDax1 (Nr0b1) | F | TGCTGCGGTCCAGGCCATCAAGAG | (1) |
| | R | GGGCACTGTTCAGTTCAGCGGATC | |
| mDnmt3l | F | CCCTCTTCCTGTATGATGATGATGG | (2) |
| | R | CCTCTGCAGCAGTCCACTCCGTGAG | |
| mDppa3 | F | GAGGACGCTTTGGATGATACAGACG | (2) |
| | R | CAACAAAGTGCGGACCCTTCTCTTG | |
| mEcat1 (Khdc3) | F | TGTGGGGCCCTGAAAGGCGAGCTGAGAT | (1) |
| | R | ATGGGCCGCCATACGACGACGCTCAACT | |
| mERas | F | ACTGCCCCTCATCAGACTGCTACT | (1) |
| | R | CACTGCCTTGTACTCGGGTAGCTG | |
| mEsrrb | F | AACCTGCCGATTTCCCCACCTGCTA | (2) |
| | R | GGCTCATCTGGTCCCCAAGTGTCAG | |
| mGapdh | F | AATGGTGAAGGTCGGTGTG | |
| | R | AAGATGGTGATGGGCTTCC | |
| emKlf4 endo | F | GGCGAGAAACCTTACCACTGT | (3) |
| | R | TACTGAACTCTCTCTCCTGGCA | |
| emcMyc endo | F | TCAAGCAGACGAGCACAAGC | (3) |
| | R | TACAGTCCCAAAGCCCCAGC | |
| emNanog endo | F | GTGCATATACTCTCTCCTTCCC | (3) |
| | R | AGCTACCCTCAAACTCCTGGT | |
| emOct3/4 endo (Pou5f1) | F | CCAACGAGAAGAGTATGAGGC | (3) |
| | R | GTGCTTTTAATCCCTCCTCAG | |
| mRex1 (Zfp42) | F | ACGAGTGGCAGTTTCTTCTTGGGA | (1) |
| | R | TATGACTCACTTCCAGGGGGCACT | |
| emSox2 endo | F | TCTGTGGTCAAGTCCGAGGC | (3) |
| | R | TTCTCCAGTTCGCAGTCCAG | |
| mUtf1 | F | GGATGTCCCGGTGACTACGTCTG | (1) |
| | R | GGCGGATCTGGTTATCGAAGGGT | |
| mZfp296 | F | CCATTAGGGGCCATCATCGCTTTC | (1) |
| | R | CACTGCTCACTGGAGGGGGCTTGC | |
| HD-dl | R | CACGGTCTTTCTTGGTAGCTG | human huntingtin mRNA (mismatch with mouse sequence) ; (4) |
| | F | CCCTGGAAAAGCTGATGAAG | |
| | | | |

| **qPCR** | | | |
|---|---|---|---|
| qmDax1 (Nr0b1) | R | ATCTGCTGGGTTCTCCACTG | (5) |
| | F | CTATCTGAAAGGGACCGTGC | |
| qmDnmt3 | R | GCTTGCTCCTGCTTCTGACT | (5) |
| | F | GGTGTGGAGCAACATTCCAG | |
| qmOct4 (Pou5f1) | R | TCTTCTGCTTCAGCAGCTTG | (5) |
| | F | GTTGGAGAAGGTGGAACCAA | |
| qmRex1 (Zfp42) | R | TATGACTCACTTCCAGGGGG | (5) |
| | F | AGAAGAAAGCAGGATCGCCT | |
| Dhcr7 | R | TGAGGTCACAGACGACCAAT | (5) |
| | F | ACAGGCCAGTCTGATGGAAG | |
| Gapdh | R | TTGATGGCAACAATCTCCAC | (5) |
| | F | CGTCCCGTAGACAAAATGGT | |
| Gpx1 | R | CAATGTAAAATTGGGCTCGAA | (5) |
| | F | GTTTCCCGTGCAATCAGTTC | |
| GSK3β | R | GTGGTTACCTTGCTGCCATC | (5) |
| | F | GACCGAGAACCACCTCCTTT | |
| H2afy | R | TGCTCACTTCTCCCTGCTTC | |
| | F | GCCAAAAAGGCCAAGTCTCC | |
| Lefty1/2 | R | TGCAGTAGACTGCTCAGGACC | (5) |
| | F | CATGAAGTCCCTGTGGCTTT | |
| Prdx1 | R | TTGATGGTATCACTGCCAGG | (5) |
| | F | CCGCTCTGTGGATGAGATTA | |
| Sod1 | R | TACTGATGGACGTGGAACCC | (5) |
| | F | GAACCATCCACTTCGAGCA | |
| Trp53 | R | TCCGACTGTGACTCCTCCAT | (5) |
| | F | CTAGCATTCAGGCCCTCATC | |

References:


1. Takahashi, K. & Yamanaka, S. Induction of pluripotent stem cells from mouse embryonic and adult fibroblast cultures by defined factors. *Cell* **126,** 663–76 (2006).
2. Yusa, K., Rad, R., Takeda, J. & Bradley, A. Generation of transgene-free induced pluripotent mouse stem cells by the piggyBac transposon. *Nat. Methods* **6,** 363–369 (2009).
3. Kaji, K. *et al.* Virus-free induction of pluripotency and subsequent excision of reprogramming factors. *Nature* **458,** 771–775 (2009).
4. Fiszer, A., Mykowska, A. & Krzyzosiak, W. J. Inhibition of mutant huntingtin expression by RNA duplex targeting expanded CAG repeats. *Nucleic Acids Res.* **39,** 5578–5585 (2011).
5. Cui, W., Taub, D. D. & Gardner, K. qPrimerDepot: a primer database for quantitative real time PCR. *Nucleic Acids Res.* **35,** D805–D809 (2007).

**SUPPLEMENTARY TABLE 2.**

Click here to Download Table S2
